# Chronic Rasmussen encephalitis presenting without seizures: A pediatric case report

**DOI:** 10.1016/j.radcr.2026.05.025

**Published:** 2026-06-03

**Authors:** Yohannes Girma Zewdie, Ermias Fikru Yesuf, Hana Yeshewas Genetirune

**Affiliations:** a Department of Radiology, Addis Ababa University, Addis Ababa, Ethiopia; b Department of Surgery, Debre Berhan University, Debre Berhan, Ethiopia; c Department of Anesthesia, Jimma University, Jimma, Ethiopia

**Keywords:** Rasmussen encephalitis, Pediatric, Cerebral atrophy, Wallerian degeneration, MRI, Atypical presentation

## Abstract

Rasmussen encephalitis is a rare chronic inflammatory neurological disorder of childhood characterized by progressive unilateral cerebral atrophy and typically associated with intractable focal seizures. We report a 6-year-old boy presenting with progressive right-sided weakness in the absence of clinically evident seizures. Brain MRI performed on a 1.5 T system demonstrated marked asymmetric atrophy of the left cerebral hemisphere, predominantly involving the insular and frontotemporal regions, with associated encephalomalacia, ex-vacuo ventricular dilatation, and ipsilateral Wallerian degeneration, without diffusion restriction or contrast enhancement. In the appropriate clinical context, these findings were suggestive of chronic Rasmussen encephalitis; however, given the absence of EEG and histopathological confirmation, this remains an imaging-based presumptive diagnosis. This case highlights an atypical presentation and emphasizes the importance of MRI in evaluating progressive focal neurological deficits even in the absence of seizures.

## Introduction

Rasmussen encephalitis is a rare, progressive, immune-mediated neurological disorder that predominantly affects children and is classically associated with drug-resistant focal epilepsy and progressive neurological decline [1,2]. Neuroimaging, particularly MRI, plays a central role in diagnosis, typically demonstrating progressive unilateral cortical atrophy and signal abnormalities [2,3]. Although seizures are considered a hallmark feature, atypical presentations without seizures have rarely been reported, posing a diagnostic challenge [4]. We present a pediatric case with imaging findings suggestive of chronic Rasmussen encephalitis presenting primarily with progressive motor deficit in the absence of seizures.

## Case presentation

A 6-year-old boy presented with gradually progressive right-sided weakness. The onset was insidious, with slow progression over time. There was no history of seizures, loss of consciousness, head trauma, central nervous system infection, or perinatal complications. Birth and developmental history were normal, and the child was fully vaccinated. Family history was noncontributory.

Neurological examination revealed right-sided motor weakness with preserved higher mental functions.

Laboratory investigations, including complete blood count, erythrocyte sedimentation rate, and C-reactive protein, were within normal limits.

Electroencephalography was not performed due to limited availability, and no clinical evidence of overt or subtle seizure activity was reported.

MRI of the brain was performed on a 1.5 Tesla system. Sequences included T1-weighted, T2-weighted, FLAIR, diffusion-weighted imaging with ADC maps, and susceptibility-weighted imaging. No intravenous contrast was administered. Slice thickness was approximately 5 mm.

Imaging demonstrated marked asymmetric volume loss of the left cerebral hemisphere, predominantly involving the insular cortex and adjacent frontotemporal regions, with cortical and subcortical encephalomalacia and gliosis. There was associated ex-vacuo dilatation of the left lateral ventricle (Figs. 1A–E). No diffusion restriction, hemorrhage, or abnormal enhancement was identified.

**Fig. 1:**
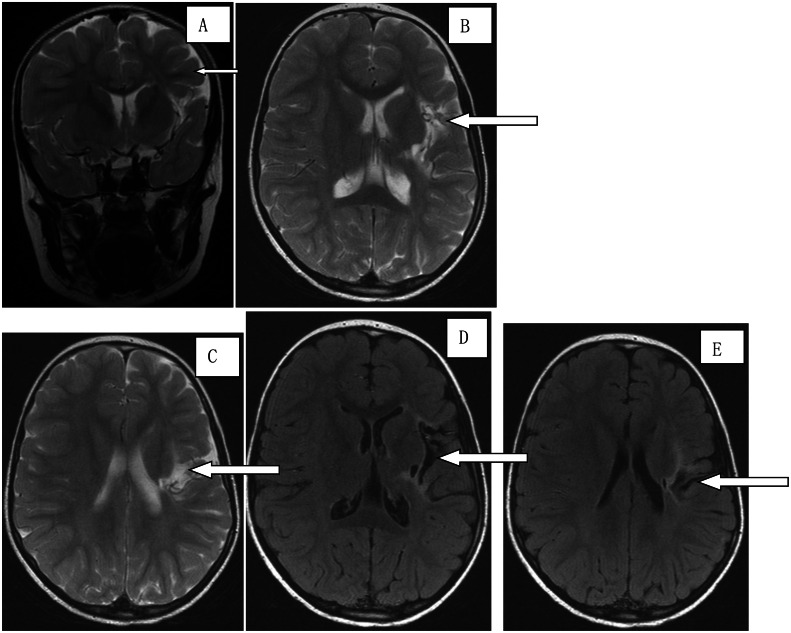
(A–E) MRI brain showing left hemispheric atrophy (white arrows) involving insular and frontotemporal regions with encephalomalacia and ex-vacuo ventricular dilatation.

There was also atrophy of the left cerebral peduncle and brainstem, consistent with Wallerian degeneration (Fig. 2) [3,5].

**Fig. 2:**
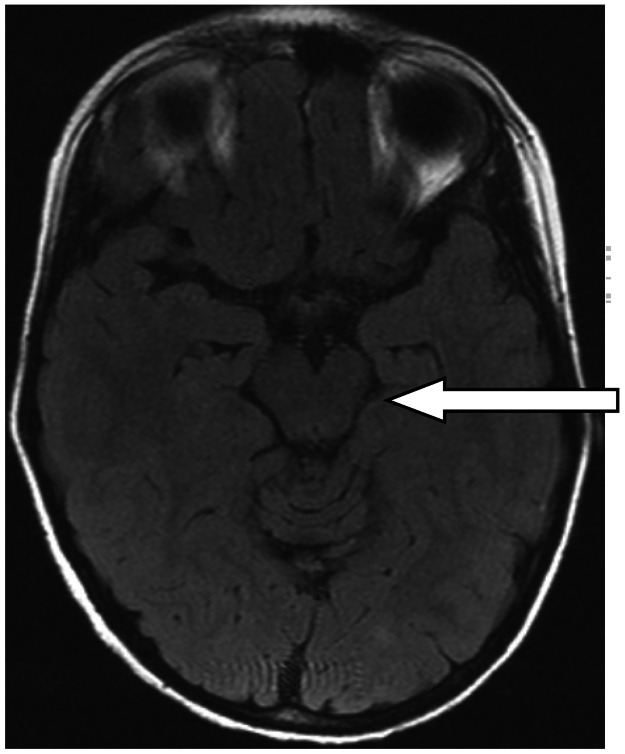
MRI demonstrating ipsilateral cerebral peduncle atrophy (white arrow), consistent with Wallerian degeneration.

The patient was managed conservatively with supportive care. On follow-up, the neurological deficit remained stable, and repeat imaging showed no significant interval change.

## Differential diagnosis

The differential diagnosis included remote left middle cerebral artery territory infarction, postinfectious encephalitis, and other causes of unilateral cerebral atrophy such as Dyke-Davidoff-Masson syndrome [6,7]. A remote infarct was considered less likely due to the lack of a clear vascular territorial pattern and progressive clinical course. Dyke-Davidoff-Masson syndrome was also unlikely given the absence of calvarial thickening, hyper-pneumatization of sinuses, and other compensatory skull changes [6].

## Discussion

Rasmussen encephalitis is characterized by progressive unilateral cerebral inflammation leading to cortical destruction, neurological decline, and typically refractory focal epilepsy [1,2]. MRI findings evolve over time, initially demonstrating cortical swelling and T2/FLAIR hyperintensity, followed by progressive hemispheric atrophy and gliosis [3,5].

This case is notable for the absence of seizures, which represents an atypical presentation. Progressive contralateral motor deficit with characteristic unilateral cortical atrophy and ipsilateral Wallerian degeneration strongly supports the imaging-based diagnosis of chronic Rasmussen encephalitis [4,5]. However, this remains a presumptive diagnosis due to the absence of EEG, CSF analysis, or histopathological confirmation.

Importantly, no imaging features of active inflammation such as cortical swelling or diffusion restriction were identified, supporting a chronic or “burnt-out” stage of disease rather than active encephalitis [3]. The stability on follow-up imaging further supports this interpretation.

MRI remains the cornerstone for diagnosis and follow-up, particularly in resource-limited settings where advanced immunological or histopathological confirmation is not feasible [2,3]. Recognition of such atypical presentations is essential to avoid misdiagnosis and unnecessary interventions.

## Conclusion

Rasmussen encephalitis should be considered in children presenting with progressive unilateral neurological deficits and characteristic unilateral cerebral atrophy on MRI, even in the absence of seizures. However, in the absence of electrophysiological or histopathological confirmation, the diagnosis should be considered presumptive. MRI remains essential for diagnosis and follow-up, particularly in resource-limited settings.

## Patient consent

Both oral and written informed consent were obtained from the patient for publication of this case report and the associated images.
